# Blood glucose upon return of spontaneous circulation and neurological outcomes following out-of-hospital cardiac arrest

**DOI:** 10.1016/j.resplu.2025.101088

**Published:** 2025-09-05

**Authors:** Ryo Yamamoto, Kazuki Matsumura, Daiki Kaito, Tomoyoshi Tamura, Koichiro Homma, Masaru Suzuki, Tomohisa Nomura, Nobuya Kitamura, Takashi Tagami, Hideo Yasunaga, Shotaro Aso, Junichi Sasaki, SOS-KANTO 2017 Study Group

**Affiliations:** a Department of Emergency and Critical Care Medicine, Keio University School of Medicine, Tokyo, Japan; b Department of Emergency Medicine, Tokyo Dental College, Ichikawa General Hospital, Chiba, Japan; c Department of Emergency and Critical Care Medicine, Juntendo University Nerima Hospital, Tokyo, Japan; d Department of Emergency and Critical Care Medicine, Kimitsu Chuo Hospital, Chiba, Japan; e Department of Emergency and Critical Care Medicine, Nippon Medical School Musashikosugi Hospital, Kanagawa, Japan; f Department of Clinical Epidemiology and Health Economics, School of Public Health, The University of Tokyo, Tokyo, Japan; g Department of Health Services Research, Graduate School of Medicine, The University of Tokyo, Tokyo, Japan

**Keywords:** Resuscitation, Critical care, Cerebral Performance Category, Neurological function

## Abstract

**Background:**

To elucidate the association between blood glucose levels at the return of spontaneous circulation (ROSC) and neurological outcomes following out-of-hospital cardiac arrest (OHCA), a post hoc analysis was conducted using data from a prospective observational study involving 41 hospitals in Tokyo and its suburbs from 2019 to 2021. Adults with nontraumatic OHCA who achieved ROSC were included.

**Methods:**

A total of 1533 patients were analyzed. A spline curve for estimating neurological outcomes (Cerebral Performance Category score ≤2 at 30 days) by blood glucose level at ROSC was generated. Patients were classified into low, moderate, and high blood glucose groups, defined as <100, 100–300, and ≥300 mg/dL, respectively. Neurological outcomes were compared using generalized estimating equations adjusted for patient and institutional characteristics.

**Results:**

Favorable neurologic outcomes were fewer in the low and high blood glucose groups than in the moderate group (8/132 [6.1 %] and 62/485 [12.8 %] vs. 181/807 [22.4 %]). The adjusted model revealed that low and high glucose levels at ROSC were associated with fewer favorable outcomes (odds ratios, 0.43 [0.18–0.96] and 0.59 [0.42–0.84]). High blood glucose levels showed unfavorable effects in subgroups with cardiogenic or noncardiogenic etiology, age <65 years, and low-flow time ≤30 min, whereas low blood glucose levels showed unfavorable effects only in cardiogenic cases.

**Conclusions:**

Blood glucose levels <100 and ≥300 mg/dL were associated with unfavorable neurological outcomes, with a nonlinear inverted U-shaped relationship.

## Highlights

• Inverted-U association existed between blood glucose at ROSC and outcomes in OHCA.
• Low and high thresholds of blood glucose at ROSC were 100 and 300 mg/dL.
• Blood glucose <100 and ≥300 mg/dL were associated with worse neurological outcomes.
• Blood glucose would potentially be used to estimate neurological functions.

## Introduction

Out-of-hospital cardiac arrest (OHCA) remains the leading cause of mortality worldwide.^1^ Despite various treatments, approximately 75 % of patients still experience unfavorable neurological outcomes.^2^, ^3^, ^4^ Therefore, discussions about continuing life-saving intensive care after return of spontaneous circulation (ROSC) often arise between healthcare providers and families, especially in cases of prolonged unconsciousness.^5^

Given the importance of accurately predicting unfavorable neurological outcomes in OHCA management, various prognostic factors have been explored.^6^, ^7^, ^8^ Studies have linked prearrest characteristics, such as older age, comorbidities, organ dysfunction, and frailty, with poor neurological outcomes despite successful ROSC.^9^, ^10^ Furthermore, factors related to cardiac arrest and resuscitation, such as witness status, bystander cardiopulmonary resuscitation (CPR), cardiac rhythm, and no–/low-flow time, are known predictors of clinical outcomes.^8^, ^11^, ^12^ As physiological status immediately after ROSC reflects the combined effects of these pre- and intra-arrest factors, its evaluation has been emphasized.^13^

Blood glucose, a key regulator of cellular metabolism, has been widely studied in critically ill patients. Glucose variability and prolonged hyper- or hypoglycemia are associated with poor outcomes. In OHCA, emerging evidence links intra-arrest glucose levels to neurological recovery after in-hospital cardiac arrest,^14^, ^15^ prehospital glycemic changes to in-hospital mortality,^16^, ^17^ and hyperglycemia to increased mortality during targeted temperature management.^18^ However, despite its potential to reflect physiological deterioration or preservation post-arrest—and its independence from later intensive care—blood glucose measured immediately after ROSC remains underexplored as a predictor of neurological outcomes in OHCA patients.

Accordingly, we conducted a post hoc analysis of a prospective, multicenter observational study of adult OHCA patients to examine the association between blood glucose levels at ROSC and neurological outcomes. We hypothesized that extreme glucose levels at ROSC would be linked to unfavorable neurological function.

## Methods

### Study design and setting

This post hoc analysis was based on a prospective, multicenter observational study conducted by the SOS-KANTO 2017 study group. It included patients with OHCA transported to 41 emergency hospitals in Tokyo and its suburbs between September 2019 and March 2021.^6^ All participating hospitals obtained institutional review board approval in accordance with the Declaration of Helsinki. The study was approved by the institutional revie board of Keio University School of Medicine on April 28, 2021 (approval number: 20210006; study title: Survey of Survivors after Cardiac Arrest in the Kanto Area 2017), with informed consent waived due to use of anonymized data.

Emergency medical service (EMS) personnel performed CPR for OHCA according to Japanese CPR guidelines, which align with the International Liaison Committee on Resuscitation and American Heart Association guidelines.^6^, ^10^ Most EMS crews include an emergency life-saving technician certified to establish intravenous access, administer medications, and use supraglottic airway devices. Endotracheal intubation can only be performed by specially trained technicians under a medical director’s instruction. Physician-staffed ambulances or helicopters, typically dispatched from tertiary care centers, are available in both urban and rural areas,^19^ though their availability varies by region. Post-ROSC intensive care in each institution followed the International Liaison Committee on Resuscitation and American Heart Association guidelines.^6^, ^10^

### Study population

This study included patients who (1) were aged ≥18 years, (2) experienced nontraumatic OHCA diagnosed by EMS and confirmed by physicians based on history and/or clinical findings, and (3) achieved ROSC. Patients without blood glucose data at ROSC were excluded.

### Data collection and definitions

Out-of-hospital OHCA data were prospectively collected by EMS providers using the standardized Utstein style, whereas in-hospital information was collected by treating physicians at each institution. Survival and neurological outcomes were assessed using the Cerebral Performance Category (CPC) scale.^20^ For patients discharged or transferred to another hospital, information needed to determine the CPC score was obtained from phone surveys.

Demographic data (age, sex, comorbidities for the Charlson Comorbidity Index, and Clinical Frailty Scale score), out-of-hospital information (place of cardiac arrest, witness status, presence of bystander CPR, initial cardiac rhythm on EMS arrival, pupillary light reflex on EMS arrival, and out-of-hospital physician presence), out-of-hospital treatment (airway device, defibrillation, epinephrine administration, and mechanical CPR use), on arrival information (cardiac rhythm, pupillary light reflex, and presence of spontaneous circulation), in-hospital information (etiology, extracorporeal membrane oxygenation, coronary angiography and revascularization, and laboratory results), and time variables (time of witness of cardiac arrest, emergency call, CPR initiation, and ROSC) were collected. Additionally, post-ROSC treatments, including intubation, extracorporeal membrane oxygenation (ECMO), coronary angiography, and revascularization, were collected. Survival status and CPC score were available at hospital discharge and 30 and 90 days after admission. Length of hospital stay was also available. Information on intra-arrest glucose administration was unavailable.

Blood glucose level at ROSC was defined as that obtained immediately after ROSC, using blood glucose levels on hospital arrival when ROSC was achieved before then. High frailty was defined as a Clinical Frailty Scale score ≥5.^21^ No-flow time was defined as the interval between the witness of cardiac arrest and CPR initiation, using the time of the emergency call as the time of cardiac arrest when the witness time was unavailable. Low-flow time was defined as the interval between CPR initiation and ROSC, using the time of ROSC at the hospital when the time of ROSC before and after hospital arrival was recorded.

### Outcome measures

The primary outcome was a favorable neurological function 30 days after admission, defined as a CPC score ≤2. The secondary outcomes included 30-day survival, favorable neurological function at discharge and after 90 days, and hospital-free days up to day 90.

### Statistical analysis

Missing nonoutcome values were replaced with a set of substituted plausible values by creating five filled-in complete data sets using multiple imputation via the chained equation method. The estimated associations in each of the imputed data sets were averaged together to obtain the overall estimated associations.^22^

Considering that a linear association between blood glucose level at ROSC and favorable neurological function at 30 days was not expected based on previous studies, restricted cubic spline curves that estimate favorable neurological function at 30 days according to blood glucose levels at ROSC were generated. Here, a generalized additive model was adopted using age, comorbidities, etiology, witness status, bystander CPR, cardiac rhythm at the scene and on arrival, prehospital medications, prehospital airway devices, prehospital physician presence, and no-flow time, after which odds ratios (ORs) for favorable neurological function at day 30 were determined according to blood glucose levels. Subsequently, thresholds of blood glucose levels at ROSC were explored.

Patients were then classified into low-, moderate-, and high blood glucose groups based on the obtained thresholds. Unadjusted analysis for differences in primary outcomes was performed using the chi-square test. Moreover, multivariable logistic regression analyses fitted with generalized estimating equations (GEEs) were performed to adjust for patient characteristics, out-of-hospital information, and resuscitation content and account for within-institution clustering.^23^ Aside from blood glucose levels, relevant covariates were selected from known or possible predictors for favorable neurologic outcomes in patients with OHCA,^1^, ^4^, ^13^, ^6^, ^7^, ^8^ including age, sex, comorbidities (Charlson Comorbidity Index), high frailty, cause of cardiac arrest (cardiogenic vs. noncardiogenic), place of cardiac arrest (home vs. public space), witness status, presence of bystander CPR, cardiac rhythm (shockable vs. nonshockable) at the scene and on hospital arrival, presence of pupillary light reflex at the scene and on arrival, out-of-hospital physician presence, out-of-hospital treatment (epinephrine, supraglottic airway device, intubation, and mechanical CPR use), and no– and low-flow time. The number of covariates in the model followed the standard upper limits for a multivariate logistic regression model (10 outcomes for each potential predictor). In the adjusted analyses, the primary outcomes between the low- and moderate blood glucose groups and between the moderate- vs. high blood glucose groups were compared.

Similarly, the secondary outcomes were examined using GEE models with the same covariates in the model for the primary outcome, during which a linear regression was fitted with the GEE model for hospital-free days. All secondary outcomes were compared between the low and moderate blood glucose groups and between the moderate and high blood glucose groups.

Five sensitivity analyses were performed to examine the robustness of the primary results. First, multivariable logistic regression analysis was performed using the same variables in the GEE model to avoid overestimating the effects of within-institution clustering. Second, inverse probability weighting analysis with propensity scores was performed,^24^ adjusting for patient and resuscitation characteristics before ROSC between the three groups. The propensity score was developed using the same variables in the GEE model, after which the effect of different blood glucose groups on the primary outcomes was examined. Third, to account for glucose variability, changes in glucose values during resuscitation were included as additional covariates, and GEE analyses were repeated. Fourth, adjustments were made using GEE models to control for confounding effects from post-ROSC treatments, such as ECMO use, coronary angiography, and revascularization. Fifth, additional covariates, diabetes as a comorbidity and dosage of epinephrine, were added to the primary model and GEE analyses were repeated.

Subgroup analyses were performed to examine the association between blood glucose levels at ROSC, clinical characteristics, and neurological outcomes. GEE analyses were repeated in patient subgroups established according to the cause of cardiac arrest (cardiogenic vs. noncardiogenic), age (<65 vs. ≥65 years), and low-flow time (≤30 vs. >30 min). The same covariates of the primary GEE models were used in most subgroup analyses, whereas multivariable logistic regression was used for subgroups with a limited sample size.

Descriptive statistics were presented as medians (interquartile ranges) or numbers (percentages). During hypothesis testing, the chi-square test and Kruskal–Wallis test were used as appropriate, with a two-sided α threshold of 0.05 indicating significance. Outcome measures were presented as OR with their 95 % confidence intervals (CIs). All statistical analyses were performed using the Statistical Package for the Social Sciences (version 29.0; IBM Corp., Armonk, NY).

## Results

### Patient characteristics and blood glucose thresholds

**Fig. 1:**
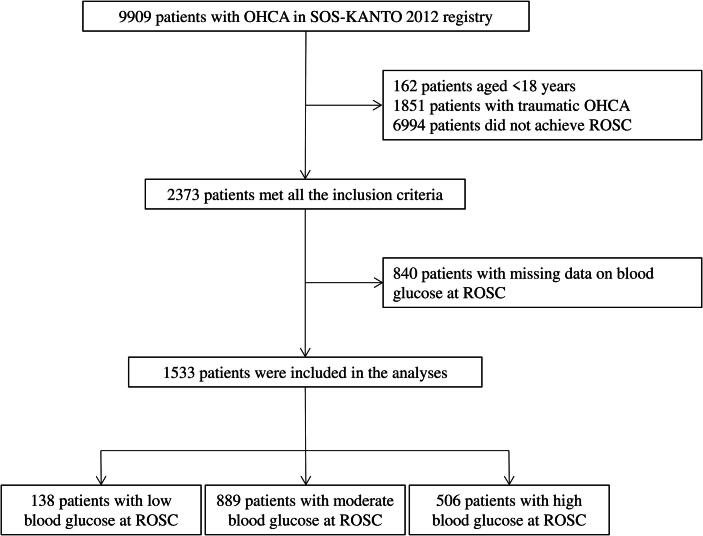
**Patient flow diagram.** Among the 9909 patients with OHCA included in the SOS-KANTO 2017 database, 1533 adult patients with nontraumatic OHCA had available blood glucose levels at ROSC and were eligible participation. A total of 138 (9.0 %), 889 (58.0 %), and 506 (33.0 %) patients had low, moderate, and high blood glucose levels at ROSC, which were defined as <100, 100–300, and ≥300 mg/dL, respectively. OHCA, out-of-hospital cardiac arrest; ROSC, return of spontaneous circulation.

**Fig. 2:**
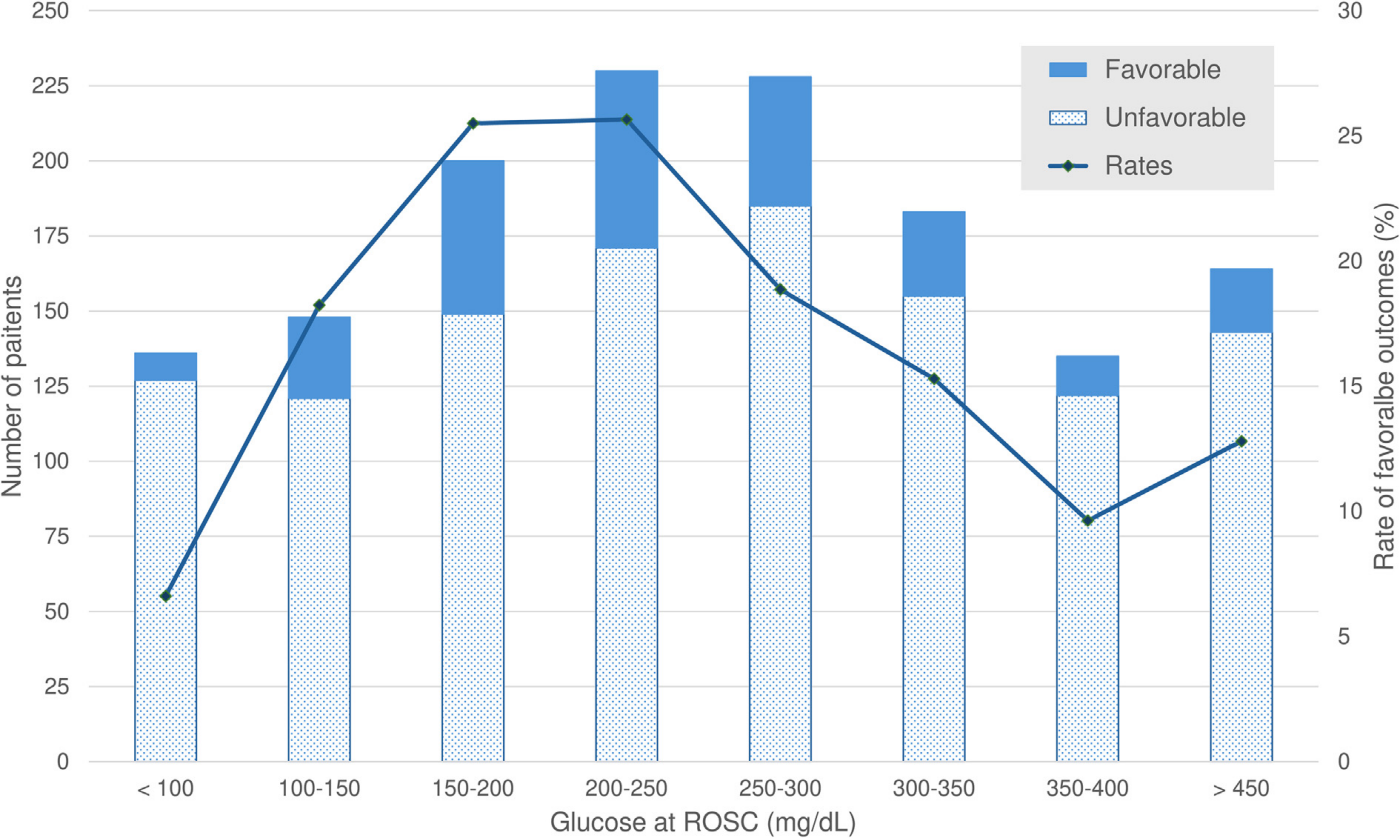
**Neurological outcomes of patients depending on blood glucose level.** The bars show the number of patients with favorable and unfavorable neurological outcomes at 30 days depending on blood glucose at ROSC, whereas the lines show the rates of favorable outcomes. Patients with blood glucose levels of 100–300 mg/dL at ROSC showed higher rates of favorable outcomes. ROSC, return of spontaneous circulation.

**Fig. 3:**
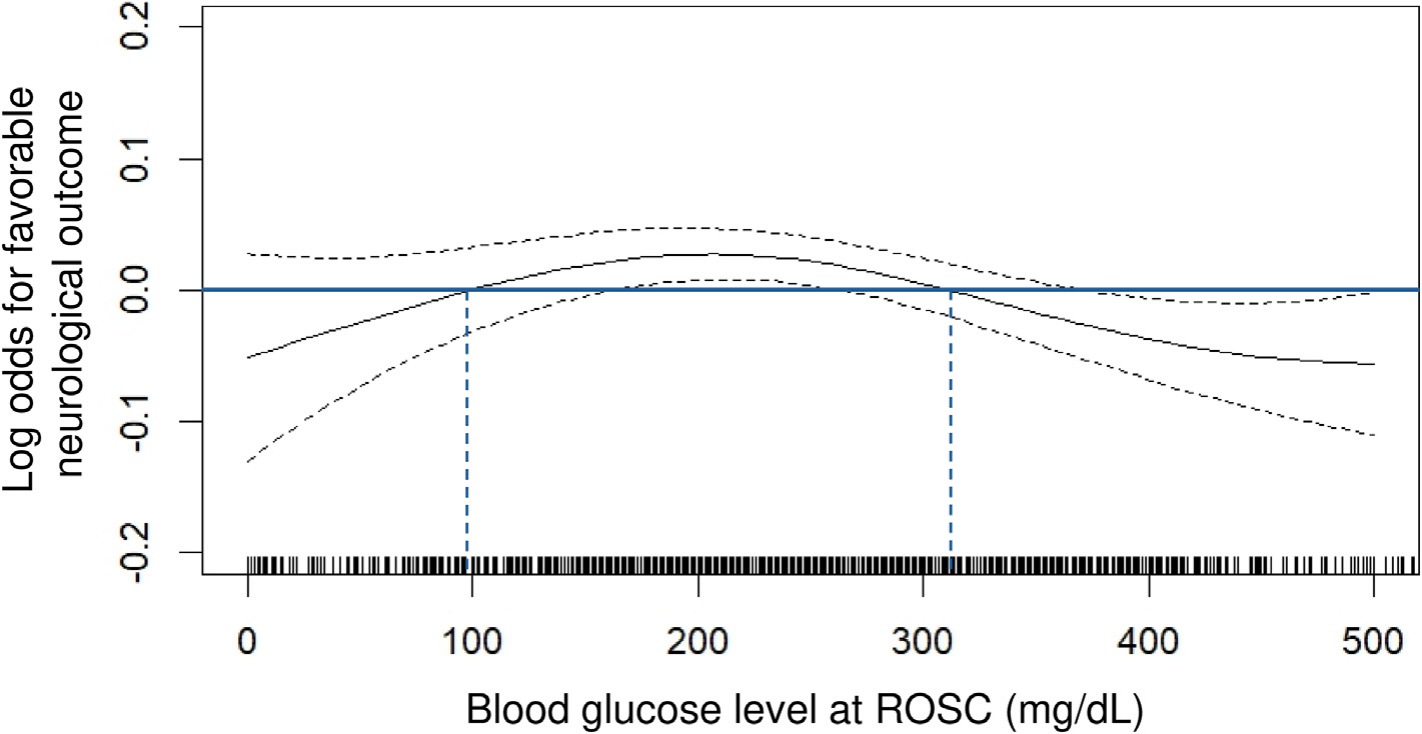
**Association between blood glucose levels and favorable neurological outcomes.** A restricted cubic spline curve illustrating the estimated probability of favorable neurological function at day 30 based on blood glucose levels at the time of ROSC is shown. Based on the curve and clinical applicability, the lower and higher blood glucose thresholds at ROSC associated with unfavorable outcomes were considered to be <100 and >300 mg/dL, respectively. ROSC, return of spontaneous circulation.

Among the 9909 patients with OHCA included in the SOS-KANTO 2017 database, 1533 adult patients with nontraumatic OHCA had available blood glucose levels at ROSC and were eligible for this study (Fig. 1). The number of patients with favorable and unfavorable neurological outcomes at day 30 depending on blood glucose at ROSC is shown in Fig. 2. Moreover, the restricted cubic spline curve for estimating favorable neurological function at day 30 according to blood glucose levels at ROSC is shown in Fig. 3. Accordingly, the low and high thresholds of blood glucose levels at ROSC for unfavorable outcomes were determined to be 100 and 300 mg/dL, respectively. Therefore, low, moderate, and high blood glucose levels at ROSC were defined as <100, 100–300, and ≥300 mg/dL, respectively.

A total of 138 (9.0 %), 889 (58.0 %), and 506 (33.0 %) patients had low, moderate, and high blood glucose levels at ROSC (Fig. 1), with their characteristics being described in Table 1. Patients with low blood glucose levels were older and had a lower albumin level than did those with moderate or high blood glucose levels. The proportion of patients with low frailty, cardiac etiology, bystander CPR, and shockable rhythm on EMS arrival and hospital arrival was lower in the low blood glucose group than in the moderate or high blood glucose group. Moreover, the proportion of patients who were male and used supraglottic airway devices was lower in the moderate blood glucose group than in the low or high blood glucose group. Furthermore, the proportion of patients who underwent coronary angiography and revascularization after ROSC was higher in the high blood glucose group than in the other two groups. The median no– and low-flow times were 9–10 and 23–28 min, respectively, between these groups.

**Table 1:** Patient characteristics of non-traumatic OHCA.

Variables		Low blood glucose		Moderate blood glucose		High blood glucose		p-value
Case		138		889		506		
Demographics								
	Blood glucose at ROSC, mg/dL, median (IQR)	60	(20–83)	210	(166–254)	369	(331–424)	
	Age, years, median (IQR)	77	(68–84)	73	(60–82)	67	(55–77)	<0.001
	Sex, male, n (%)	49	(35.5 %)	243	(27.3 %)	192	(37.9 %)	<0.001
	Comorbidity, Charlson index, median (IQR)	0	(0–1)	0	(0–1)	0	(0–1)	0.003
	High frailty*, n (%)	60	(43.5 %)	261	(29.4 %)	107	(21.1 %)	<0.001
	Etiology, cardiogenic, n (%)	69	(50.0 %)	603	(67.8 %)	327	(64.6 %)	<0.001
Prehospital information, n (%)								
	Cardiac arrest at home	138	(100.0 %)	889	(100.0 %)	506	(100.0 %)	−
	Witness presence	89	(64.5 %)	621	(69.9 %)	357	(70.6 %)	0.295
	Bystander CPR	57	(41.3 %)	462	(52.0 %)	268	(53.0 %)	0.042
	Shockable rhythm on EMS arrival**	13	(9.4 %)	199	(22.4 %)	148	(29.2 %)	<0.001
	No pupillary light reflex	125	(90.6 %)	770	(86.6 %)	461	(91.1 %)	0.066
	Cardiac arrest on route	18	(13.0 %)	91	(10.2 %)	73	(14.4 %)	0.060
Prehospital treatment, n (%)								
	Supraglottic airway device	64	(46.4 %)	315	(35.4 %)	214	(42.3 %)	0.006
	Intubation	17	(12.3 %)	115	(12.9 %)	84	(16.6 %)	0.137
	Epinephrine	69	(50.0 %)	339	(38.1 %)	222	(43.9 %)	0.009
	Mechanical CPR	12	(7.1 %)	63	(7.1 %)	43	(8.5 %)	0.572
	Prehospital physician presence	14	(10.1 %)	114	(12.8 %)	75	(14.8 %)	0.303
Information on hospital arrival, n(%)								
	Shockable rhythm on arrival**	7	(5.1 %)	58	(13.8 %)	70	(13.8 %)	<0.001
	No pupillary light reflex	118	(85.5 %)	657	(73.9 %)	432	(85.4 %)	<0.001
No-flow time, min, median (IQR)		9	(6–13)	10	(6–13)	9	(6–13)	0.580
Low-flow time, min, median (IQR)		27	(16–36)	23	(10–35)	28	(18–41)	<0.001
Treatment after ROSC, n (%)								
	ECMO	2	(1.4 %)	36	(4.0 %)	34	(6.7 %)	0.013
	Coronary angiography	11	(8.0 %)	273	(30.7 %)	188	(37.2 %)	<0.001
	Coronary revascularization	4	(2.9 %)	123	(13.8 %)	105	(20.8 %)	<0.001
Laboratory on admission, median (IQR)								
	PaO_2_, mmHg	152	(87–290)	126	(67–267)	159	(81–315)	<0.001
	PaCO_2_, mmHg	55	(37–82)	49	(36–75)	59	(41–83)	<0.001
	Base excess, mEq/L	−15	(−21- −5)	−11	(−17- −5)	−18	(–22- −12)	<0.001
	WBC, 10^3^/μL	9.7	(6.7–12.5)	10.4	(7.9–13.3)	11.1	(8.2–14.7)	<0.001
	Hemoglobin, g/dL	11.4	(9.8–13.2)	12.3	(10.5–14.1)	12.5	(10.6–14.1)	0.002
	Na, mEql/L	140	(137–143)	140	(137–143)	140	(137–143)	0.939
	K, mEql/L	5.5	(4.4–6.6)	4.4	(3.7–5.5)	4.4	(3.7–5.4)	<0.001
	Albumin, g/dL	2.8	(2.4–3.4)	3.3	(2.7–3.8)	3.3	(2.9–3.6)	<0.001

Low, moderate, high blood glucose were defined as <100, 100–300, >=300 mg/dL, respectively. OHCA, out-of-hospital cardiac arrest; IQR, interquartile range; CPR, cardiopulmonary resuscitation; EMS, emergency medical services; ROSC = return of spontaneous circulation; ECMO, extracorporeal membrane oxygenation; and WBC, white blood cell count.

* High frailty was defined as Clinical Frailty Scale >=5.

** Shockable rhythm includes ventricular fibrillation and pulseless ventricular tachycardia.

### Favorable neurological outcomes and secondary outcomes

The rate of favorable neurologic function 30 days after ROSC from OHCA was significantly lower in the low (8 [6.1 %]) and high (62 [12.8 %]) blood glucose groups at ROSC than in the moderate blood glucose group (181 [22.4 %]) on unadjusted analysis (*p* < 0.001; OR, 0.22 [95 % CI, 0.11–0.46] and 0.51 [0.37–0.69] for low and high to moderate blood glucose levels, respectively; Table 2).

**Table 2:** Neurological and other clinical outcomes.

Outcomes	Low blood glucose	Moderate blood glucose	High blood glucose	p value	Log blood glucose*			High blood glucose*		
					OR (95 % CI)		p value	OR (95 % CI)		p value
Cerebral performance category <=2 at 30 days										
− unadjusted, *n/total (%)*	8/132 (6.1 %)	181/807 (22.4 %)	62/485 (12.8 %)	<0.001	0.22	(0.11 to 0.46)		0.51	(0.37 to 0.69)	
− adjusted with GEE model					0.43	(0.18 to 0.96)	0.041	0.59	(0.42 to 0.84)	0.004
− adjusted with logistic regression model					0.53	(0.33 to 0.86)	0.010	0.42	(0.29 to 0.63)	<0.001
− adjusted with IPW, *(%)*	5.0 %	22.2 %	14.2 %	<0.001	0.18	(0.14 to 0.25)		0.58	(0.48 to 0.70)	
30-day survival, *n/total (%)*	29/135 (21.5 %)	302/846 (35.7 %)	128/495 (25.9 %)		0.85	(0.57 to 1.27)	0.423	0.61	(0.49 to 0.77)	<0.001
Cerebral performance category <=2 at discharge, *n/total (%)*	8/137 (5.8 %)	217/867 (25.0 %)	68/491 (13.8 %)		0.34	(0.16 to 0.74)	0.007	0.56	(0.37 to 0.84)	0.005
Cerebral performance category <=2 at 90 days, *n/total (%)*	7/125 (5.6 %)	174/775 (22.5 %)	56/461 (12.1 %)		0.36	(0.14 to 0.88)	0.025	0.53	(0.35 to 0.80)	0.002
Hospital-free-days up to 90 day, *days, mean, median (IQR)*	10, 0 (0–0)	23, 0 (0–60)	12, 0 (0–0)		−4	(−10 to 1)**	0.083	−7	(−10 to −4)**	<0.001

Low, moderate, high blood glucose were defined as <100, 100–300, >=300 mg/dL, respectively. OR, odds ratio; CI, confidence interval; GEE, generalizing estimating equation; IPW, inverse probability weighting; and IQR, interquartile range.

* Reference was moderate blood glucose.

** Increase in number of days was shown.

The GEE model for adjusted OR developed with OHCA- and resuscitation-related covariates (not with post-ROSC variables) showed that low and high blood glucose levels at ROSC was associated with a lower rate of favorable neurologic function at 30 days (adjusted OR, 0.43 [0.18–0.96]; *p* = 0.041 and OR, 0.59 [0.42–0.84]; *p* = 0.004 for low and high to moderate blood glucose levels, respectively; Table 2). Moreover, all sensitivity analyses similarly revealed fewer favorable neurological outcomes in the low and high blood glucose groups at ROSC than in the moderate blood glucose group, regardless of institutional differences in managements, glucose variability during resuscitation, and post-ROSC treatments (Table 2 and Supplemental Table S1).

Lower incidences of favorable neurological function (CPC score ≤2) at hospital discharge and at 90 days after ROSC were also associated with low and high blood glucose at ROSC (8 [5.8 %], 217 [25.0 %], and 68 [13.8 %] at discharge in the low, moderate, and high blood glucose group; OR, 0.34 [0.16–0.74] and 0.56 [0.37–0.84] for low and high to moderate blood glucose and 7 [5.6 %], 174 [22.5 %], and 56 [12.1 %] at 90 days in the low, moderate, and high blood glucose group; OR, 0.36 [0.14–0.88] and 0.53 [0.35–0.80] for low and high to moderate blood glucose levels; Table 2). Conversely, significant differences in 30-day survival and hospital-free days up at 90 days were only observed between the moderate and high blood glucose groups at ROSC.

### Subgroup analysis

Subgroup analyses (Table 3) revealed a relationship between blood glucose at ROSC and unfavorable neurological function at 30 days. In particular, a high blood glucose level was associated with lower rates of favorable neurological outcomes in patients with cardiogenic or noncardiogenic etiology, nonelderly adults (<65 years), and those with low-flow time ≤30 min (OR, 0.58 [0.36–0.96], 0.66 [0.66–0.66], 0.34 [0.18–0.62], and 0.47 [0.30–0.72] compared to a moderate blood glucose level, respectively; Table 3). A low blood glucose level was associated with unfavorable neurological outcomes only in patients with a cardiogenic etiology (OR, 0.37 [0.14–0.93]; Table 3).

**Table 3:** Favorable neurological outcome in subgroup analyses.

Subgroups		Low blood glucose	Moderate blood glucose	High blood glucose	OR (95 % CI)			
					Log blood glucose*		High blood glucose*	
Etiology								
	Cardiogenic	5/68 (7.4 %)	164/539 (30.4 %)	57/310 (18.4 %)	**0.37**	**(0.14 to 0.93)**	**0.58**	**(0.36 to 0.96)**
	Non-cardiogenic	3/64 (4.7 %)	17/268 (6.3 %)	5/175 (2.9 %)	0.62	(0.14 to 2.69)	**0.66**	**(0.66 to 0.66)**
Age								
	< 65 years	3/28 (10.7 %)	93/241 (38.6 %)	34/214 (15.9 %)	0.54	(0.15 to 1.92)	**0.34**	**(0.18 to 0.62)**
	>= 65 years	5/104 (4.8 %)	88/566 (15.5 %)	28/271 (10.3 %)	0.41	(0.12 to 1.33)	0.94	(0.62 to 1.44)
Low-flow time								
	<=30 min	4/55 (7.3 %)	120/390 (30.8 %)	36/205 (17.6 %)	0.31	(0.07 to 1.31)	**0.47**	**(0.30 to 0.72)**
	>30 min	1/40 (2.5 %)	6/217 (2.8 %)	11/174 (6.3 %)	0.58	(0.13 to 2.55)	0.81	(0.08 to 7.81)

* Low, moderate, high blood glucose were defined as <100, 100–300, >=300 mg/dL, respectively. Number (%) of patients with Cerebral Performance Category <=2 at 30 days was shown. Generalizing estimating equations were performed in each subgroup except for low-flow time >30 min. OR, odds ratio and CI, confidence interval. Reference was moderate blood glucose.

Patients aged ≥65 years and those with low-flow time >30 min had a comparable incidence of favorable neurological outcomes regardless of blood glucose levels at ROSC.

## Discussion

This study demonstrated that low (<100 mg/dL) and high (≥300 mg/dL) blood glucose levels at ROSC were associated with unfavorable neurological outcomes in patients resuscitated from nontraumatic OHCA, even after adjusting for patient background, resuscitation characteristics, institutional factors, glucose variability, and post-ROSC treatments.

Several pathophysiological mechanisms behind the unfavorable neurological outcomes in patients with low or high blood glucose at ROSC should be considered. First, hypoglycemia immediately after ROSC could indicate energy storage depletion, which could impede recovery from multiple organ dysfunction following cardiac arrest.^25^, ^26^ Given that resuming cell metabolism in vital tissues requires optimal generation of adenosine triphosphate by primarily utilizing glucose, low blood glucose levels at ROSC could be associated with unsuccessful restoration of disturbed organ functions. Notably, low blood glucose levels were also associated with lower survival at 30 days, suggesting that hypoglycemia affected various organs aside from the brain. Second, low blood glucose levels after resuscitation could be a surrogate marker of abnormalities in glycogenesis, glycogenolysis, and/or insulin response. Previous studies have observed spontaneous hypoglycemia in certain patients with critical illnesses, such as hepatic failure, acute kidney disease, hormonal deficiency, and other inflammatory diseases.^27^, ^28^ Therefore, low blood glucose levels could reflect severe malfunctioning of homeostasis due to cardiac arrest.

Third, high blood glucose levels could also be associated with a higher degree of illness severity or inflammation following ROSC. This phenomenon, commonly observed among critically ill patients, is known as stress hyperglycemia, a physiological response coordinated by the hypothalamic–pituitary–adrenal axis and the sympathoadrenal system.^29^ Indeed, previous studies have found a relationship between stress hyperglycemia in intensive care units and neurologic outcome in patients with post-cardiac arrest syndrome.^18^, ^30^ Although no study has thoroughly elucidated the direct influence of hyperglycemia on neurological outcomes, high blood glucose levels at ROSC could be an early indication of stress hyperglycemia during intensive care. Finally, patients with high blood glucose levels at ROSC could have undiagnosed comorbidities, including atherosclerosis-related diseases.^31^ Although the current analysis adjusted for patient backgrounds, such as coronary artery diseases and cerebral vascular diseases, only diagnosed comorbidities could be statistically controlled. Preexisting, undiagnosed, and undertreated atherosclerosis-related diseases could have affected neurological functions following OHCA in patients with high blood glucose levels at ROSC.

Subgroup analyses showed that older patients (≥65 years) and those with prolonged low-flow time (>30 min) had similar clinical outcomes regardless of blood glucose level at ROSC, suggesting that the relatively low effects of hypo- or hyperglycemia would not have emerged in patients with high baseline risks for devastating neurological outcomes. Furthermore, low blood glucose levels at ROSC have been associated with unfavorable neurological functions only in patients with cardiogenic etiology, which should be interpreted cautiously given the limited sample size and a few outcome events.

Notably, this study focused on blood glucose levels immediately after ROSC because blood glucose would have been managed thereafter following standard thresholds, such as 110 to 180 mg/dL. Therefore, the glucose thresholds identified in this study (100 and 300 mg/dL) will only be useful to predict neurological outcomes at the time of ROCS, not for the critical care targets following ROSC. In addition, the higher threshold of glucose in this study (300 rather than 180 mg/dL) would support the idea that a pathophysiological glucose metabolism will differ between intra-arrest resuscitation and post-ROSC intensive care. Considering that a recent study indicated the standard blood glucose management was not associated with survival after ROSC in patients with OHCA,^32^ the new cut-offs designated for post-ROSC care need to be further examined. Moreover, the glucose level of 100 –300 mg/dL at ROSC can be utilized for the discussions of continuing life-saving intensive care, whereas the clinical utility as a single indicator for favorable outcomes should be further analyzed.

### Limitations

Our results should be interpreted in light of the study design. First, the study retrospectively used data from the SOS-KANTO study, which lacked information on treatments for low and high blood glucose levels before ROSC. Thus, unrecorded prognostic factors, such as resuscitation quality and glucose administration, could have influenced outcomes. Particularly, under-resuscitation without appropriate prehospital glucose administration would introduce low blood glucose at ROSC and unfavorable neurological outcomes. Second, data on organ-specific pathophysiological changes, including hemodynamic status and cerebral oxygenation, were unavailable. Although suboptimal recovery may have contributed to unfavorable outcomes in patients with low or high glucose, this could not be objectively confirmed. The lack of cortisol and insulin levels also could not confirm physiological mechanisms of the current results. Third, because only patients who achieved ROSC were included, no conclusions can be drawn about intra-arrest glucose and resuscitation success. Lastly, the study did not assess interventions targeting glucose levels between 100 and 300 mg/dL.

## Conclusions

This study revealed a nonlinear, inverted U-shaped relationship between blood glucose at ROSC and 30-day neurological outcomes in patients resuscitated from nontraumatic OHCA, with both low (<100 mg/dL) and high (≥300 mg/dL) levels linked to unfavorable outcomes.

## Consent for publication

Not applicable.

## Availability of data and material

The data used in this study are available from the SOS-KANTO 2017 study group. However, access to these data is restricted, as they were used under license for the current study and are not publicly available. Data may be obtained from the authors upon reasonable request and with permission from the SOS-KANTO 2017 study group.

## Authors’ contributions

RY, KM, DK, and KH designed the study. TN, NK, and TT performed data collection. HY, SA, and JS managed quality control. RY, DK, and KH performed data analysis. RY, DK, TT, and JS contributed to data interpretation, manuscript writing, and critical revision. All authors read and approved the final manuscript.

## CRediT authorship contribution statement

**Ryo Yamamoto:** Writing – review & editing, Writing – original draft, Validation, Methodology, Formal analysis, Data curation, Conceptualization. **Kazuki Matsumura:** Writing – review & editing, Methodology, Conceptualization. **Daiki Kaito:** Writing – review & editing, Methodology, Investigation, Conceptualization. **Tomoyoshi Tamura:** Writing – review & editing, Data curation. **Koichiro Homma:** Writing – review & editing, Methodology, Conceptualization. **Masaru Suzuki:** Writing – review & editing, Methodology, Data curation. **Tomohisa Nomura:** Writing – review & editing, Data curation. **Nobuya Kitamura:** Writing – review & editing, Data curation. **Takashi Tagami:** Writing – review & editing, Data curation. **Hideo Yasunaga:** Writing – review & editing, Validation, Methodology. **Shotaro Aso:** Writing – review & editing, Methodology. **Junichi Sasaki:** Writing – review & editing, Supervision.

## Ethics approval and consent to participate

This study was approved by the Institutional Review Board of Keio University School of Medicine on April 28, 2021 (approval number: 20210006; study title: Survey of Survivors after Cardiac Arrest in the Kanto Area 2017).

## Funding

This research did not receive any specific grant from funding agencies in the public, commercial, or not-for-profit sectors.

## Declaration of competing interest

The authors declare that they have no known competing financial interests or personal relationships that could have appeared to influence the work reported in this paper.

## Glossary

CI: confidence interval
CPC: Cerebral Performance Category
GEE: generalized estimating equation
OR: odds ratio
