# Supplementary material for: Blood glucose upon return of spontaneous circulation and neurological outcomes following out-of-hospital cardiac arrest
Source: Resusc Plus. 2025 Sep 5;26:101088. doi: 10.1016/j.resplu.2025.101088 (PMC12481122; doi:10.1016/j.resplu.2025.101088)
Supplement: Supplementary Data 1 [file mmc1.docx]

**Supplemental Table S1. Sensitivity analyses using other GEE models**

|  | | | | |  |
| --- | --- | --- | --- | --- | --- |
| Type of analysis | Log blood glucose* | | High blood glucose* | |  |
|  | OR (95% CI) | | OR (95% CI) | |  |
| Cerebral performance category <=2 at 30 days |  |  |  |  |  |
| - adjusted with glucose variation | 0.07 | (0.01 to 0.59) | 0.59 | (0.41 to 0.83) |  |
| - adjusted with post-ROSC treatment | 0.34 | (0.01 to 0.58) | 0.59 | (0.42 to 0.84) |  |
| - adjusted with diabetes and epinephrine dosage | 0.35 | (0.12 to 0.99) | 0.55 | (0.38 to 0.79) |  |
| Low, moderate, high blood glucose were defined as <100, 100-300, >=300 mg/dL, respectively. OR, odds ratio; CI, confidence interval; and ROSC, return of spontaneous circulation. *Reference was moderate blood glucose. | | | | |  |
|  |  |  |  |  |  |
|  |  |  |  |  |  |
